# Teachers’ Emotional Exhaustion: Associations With Their Typical Use of and Implicit Attitudes Toward Emotion Regulation Strategies

**DOI:** 10.3389/fpsyg.2020.00867

**Published:** 2020-05-29

**Authors:** Monika H. Donker, Marja C. Erisman, Tamara van Gog, Tim Mainhard

**Affiliations:** Department of Education, Utrecht University, Utrecht, Netherlands

**Keywords:** teachers, emotional exhaustion, emotion regulation, expressive suppression, cognitive reappraisal, Implicit Association Test

## Abstract

Teaching is an emotionally challenging profession, sometimes resulting in high levels of teacher stress, burnout, and attrition. It has often been claimed that certain emotion regulation strategies can lower teachers’ feelings of burnout. The use of cognitive reappraisal (i.e., cognitively changing the emotional impact of a situation) has generally been associated with positive outcomes, whereas using expressive suppression (i.e., inhibiting emotional responses) usually has negative consequences. The present study investigated the association between teachers’ typical use of these two emotion regulation strategies (i.e., cognitive reappraisal and expressive suppression) and their feelings of emotional exhaustion. Because there is evidence that regulating emotions could involve higher costs when regulation goes against individual preferences, we also explored the potentially moderating effect of teachers’ implicit attitudes toward emotion regulation versus emotion expression on the association between typical use of emotion regulation strategies and teachers’ emotional exhaustion with an Implicit Association Test (IAT). We included the interpersonal teacher–student relationship (in terms of teacher agency and communion), teacher experience, and teacher gender as covariates in our analyses. Participants were 94 teachers in secondary education, vocational education, and teacher training for secondary education. Replicating findings from prior studies, hierarchical regression analyses showed that typical use of cognitive reappraisal, but not expressive suppression, was significantly related to lower levels of teachers’ emotional exhaustion. Teachers’ implicit attitudes toward emotion regulation versus emotion expression moderated the relationship between the use of emotion regulation strategies and emotional exhaustion, but only in a subsample with more experienced teachers. Teachers who showed more interpersonal agency in class and had more years of teaching experience reported lower levels of emotional exhaustion. Interpersonal communion and gender were not directly associated with feelings of exhaustion. Implications for teacher training and suggestions for future research are discussed.

## Introduction

Teaching is a challenging profession, resulting in high levels of burnout among teachers (EU-OSHA, 2013). Next to administrative workload (Farber, 1984; Van Droogenbroeck et al., 2014), a problematic teacher–student relationship is a well-known contributor to negative emotions, decreased work engagement, and increased emotional exhaustion (Spilt et al., 2011; Klassen et al., 2012; Aldrup et al., 2018). It has been suggested that the use of appropriate emotion regulation strategies might help to prevent feelings of burnout (Cross and Hong, 2012; Durr et al., 2014; Keller et al., 2014) and feelings of emotional exhaustion in particular (Tsouloupas et al., 2010). Most previous studies found that, in general, using cognitive reappraisal strategies (i.e., cognitively changing the emotional impact of a situation) yields more positive emotions, better interpersonal functioning, and higher levels of well-being, whereas using expressive suppression (i.e., inhibiting emotional responses) might result in more negative outcomes (Gross and John, 2003; Moore et al., 2008; Haga et al., 2009; Webb et al., 2012). However, regulating emotions by means of emotion regulation strategies is effortful and could involve higher costs when regulation goes against an individual’s implicit preference for emotion regulation versus emotion expression. For example, teachers often feel there are certain emotional display rules they need to adhere to (e.g., not expressing your anger toward student misbehavior). The extent to which an individual’s implicit positive attitude toward emotion regulation (as opposed to emotion expression) is aligned with those display rules might affect the costs and benefits associated with the use of explicit emotion regulation strategies (Chang and Davis, 2009; Frenzel, 2014).

Little is known about the interplay of teachers’ typical use of and their implicit attitudes toward emotion regulation strategies. Studying this interplay could help to further specify the ways in which we can support teachers in dealing with unpleasant emotions, which might ultimately lead to lower levels of teacher stress and emotional exhaustion. Therefore, the present study investigated not only the association between teachers’ typical use of emotion regulation strategies and their feelings of emotional exhaustion but also the potential moderating role of their implicit attitudes toward emotion regulation versus emotion expression. Moreover, other factors that have been related to teacher’s emotional exhaustion were taken into account, namely, the quality of the teacher–student relationship (Chang, 2009; Spilt et al., 2011), years of teaching experience (Grandey, 2000; Harmsen et al., 2018), and teacher gender (Johnson and Spector, 2007; Olson et al., 2019).

Teacher burnout has received a considerable amount of attention, both from policy makers as well as in educational research. Burnout can be defined as “a prolonged response to chronic emotional and interpersonal stressors on the job, and is defined by the three dimensions of emotional exhaustion, cynicism, and inefficacy” (Maslach et al., 2001, p. 397). Emotional exhaustion is often considered to be the most central aspect of burnout and has been the focus of attention in many studies in the educational context (Chang, 2009; Tsouloupas et al., 2010; Goetz et al., 2015; Arens and Morin, 2016; Taxer et al., 2019). Moreover, it has been suggested that the use of effortful emotion regulation strategies might in particular affect employees’ feelings of emotional exhaustion because of the emotional dissonance and emotional labor they experience when applying regulation strategies (Johnson and Spector, 2007; Keller et al., 2014).

Already in the 1980s, studies indicated high levels of stress experienced by teachers, in some cases leading to burnout (Farber, 1984; Kyriacou, 1987). Since the introduction of the Maslach Burnout Inventory (MBI; Maslach et al., 1996), research on burnout has rapidly increased. Although such research efforts have improved our understanding of the phenomenon, recent papers indicate that teacher burnout is still frequent and has long-lasting consequences for both teachers and their students, such as high turnover and dropout rates among teachers and lower achievement and school satisfaction among students (Arens and Morin, 2016; Veldman et al., 2016; Lee, 2019). Daily emotions have often been described as the “building blocks” of burnout (Hollenstein, 2015), and the regulation of emotions and personal preferences regarding emotion regulation have been proposed to play an important role in personal well-being and social functioning (Sutton and Wheatley, 2003; Jiang et al., 2016).

Two of the most well-known emotion regulation strategies are cognitive reappraisal and expressive suppression (Gross and John, 2003; Moore et al., 2008). *Cognitive reappraisal* is an emotion regulation strategy aiming at explicitly and cognitively changing ones thoughts and behavior before an emotion has fully developed (also referred to as antecedent-focused or deep-acting strategies; Lazarus and Alfert, 1964; Gross and John, 2003; Lee et al., 2016). In the case of teaching, a teacher might, for example, choose to label the situation in which a student talks to another student during plenary instruction as a sign of interest rather than disengagement and thus experience positive instead of negative emotions. Cognitive reappraisal has been found to be the emotion regulation strategy with the best well-being outcomes for teachers and has therefore been considered effective (e.g., Gross and John, 2003; Barber et al., 2011; Becker et al., 2015; Jiang et al., 2016; Lavy and Eshet, 2018). However, some studies in other contexts have also indicated potential negative effects of cognitive reappraisal. For example, there is some evidence that cognitive reappraisal only has positive effects when used in uncontrollable situations (i.e., where you can only control yourself, not the environment), but negative effects in situations where participants reported that they could have influenced the stressful situation (Troy et al., 2013). Haines et al. (2016) also found evidence for what has been called the *strategy-situation-fit hypothesis* and the importance of regulatory flexibility (Bonanno and Burton, 2013) using an experience sampling design. They found that participants who were using cognitive reappraisal only in uncontrollable situations scored higher on well-being (Haines et al., 2016). This suggests that during teaching, which could be characterized as a (mostly) controllable situation for teachers, the effects of using cognitive reappraisal strategies might be limited or even negative.

*Expressive suppression*, on the other hand, aims at regulating the expression of an emotion that is already experienced (also referred to as response-focused or surface-acting strategies; Gross, 1998; Gross and John, 2003; Lee et al., 2016). Expressive suppression frequently occurs especially in situations where others are present and where the goal is to avoid conflict (English et al., 2017). For example, teachers may choose to suppress their anger and stay friendly in case of disrupting student behavior to avoid escalation or discussion with students. Expressive suppression has often been related to negative outcomes for teachers such as an increase in stress-related symptoms and emotional exhaustion (Butler et al., 2003; Moore et al., 2008; Haga et al., 2009; Barber et al., 2011; Jiang et al., 2016). However, there are studies in other contexts that showed positive effects of expressive suppression strategies, for example, in the context of combatting food cravings (Siep et al., 2012). Moreover, Richardson (2017) showed with a daily-diary study that using expressive suppression only had negative effects on affective well-being on high-stress days, but not on low-stress days where emotion regulation might be less costly. Also, in the teaching context, it has been suggested that expressive suppression of negative emotions may be functional because it is in line with common standards that guide appropriate expression of emotions in the classroom (i.e., display rules; Hagenauer and Volet, 2014; Taxer and Frenzel, 2015).

In sum, both cognitive reappraisal and expressive suppression may have benefits as well as costs. Negative effects are often explained by the effort required from the teacher to exert emotion regulation strategies in general, which potentially impairs not only cognitive performance (Richards and Gross, 1999) and achievement (Low et al., 2017) but also peer relationships (Tsai et al., 2017) and social interactions (Butler et al., 2003). In other words, teachers may pay a price for using effortful emotion regulation strategies. Although regulating your emotions may help on the short term, the effort teachers put into it may have negative consequences in the long run. In the teaching situation, the cognitive resources absorbed by teachers’ use of effortful emotion regulation strategies might result in less available resources for classroom management and the actual teaching itself. Moreover, when teachers feel compelled to use emotion regulation strategies imposed by school leaders, parents, or society, this might have negative consequences for their well-being (Hagenauer and Volet, 2014; Taxer and Frenzel, 2015). Therefore, the use of emotion regulation strategies has also been referred to as *emotional labor* (Hochschild, 1983).

In line with Frenzel’s theoretical model on teacher emotions (Frenzel, 2014), it could be argued that teachers may view emotional labor as a positive part of their job. Implicit beliefs, habits, and cultural norms may help teachers to internalize an implicit positive attitude toward the use of emotion regulation strategies (Sutton and Harper, 2009; Sutton et al., 2009). Having such an implicit positive attitude toward emotion regulation (as opposed to emotion expression) has been proposed to be beneficial for the effort involved in the actual use of emotion regulation strategies and well-being outcomes (Koole and Jostmann, 2004; Mauss et al., 2006). An implicit positive attitude toward emotion regulation might lower the cognitive and emotional costs of the use of effortful emotion regulation strategies (Mauss et al., 2007). Mauss et al. (2006) developed an Implicit Association Test (IAT) to examine implicit attitudes toward emotion regulation (as opposed to emotion expression) in addition to self-reported use of emotion regulation strategies. Participants who had an implicit positive attitude toward emotion regulation (as opposed to emotion expression) reported less anger and fewer negative thoughts after an anger provocation task that required emotion regulation. In a follow-up study, Hopp et al. (2011) found support for their hypothesis that having an implicit positive attitude toward emotion regulation might only increase psychological health when adaptive emotion regulation strategies, such as cognitive reappraisal, are used, but not when using expressive suppression of emotions.

Thus, while using cognitive reappraisal strategies might help teachers to effectively deal with their emotions and diminish their feelings of emotional exhaustion, these strategies may also be experienced as effortful, depending on whether teachers do or do not have an implicit positive attitude toward emotion regulation (as opposed to emotion expression). Similarly, when using expressive suppression, an implicit positive attitude toward emotion regulation may be beneficial because it may help teachers to suppress their emotions more easily, and the cognitive and emotional costs of suppression might therefore be lower.

The present study builds on the existing literature by investigating the association between teachers’ typical use of emotion regulation strategies and emotional exhaustion and by exploring whether teachers’ implicit attitude toward emotion regulation (as opposed to emotion expression) had a moderating effect on this association using an IAT. Two main research questions and hypotheses guided our investigation:

1. To what extent is teachers’ typical use of emotion regulation strategies associated with their level of emotional exhaustion?

In line with previous research, we expected that teachers’ use of cognitive reappraisal strategies and their emotional exhaustion level would be negatively associated (i.e., more use of cognitive reappraisal is associated with lower levels of emotional exhaustion), while using expressive suppression would be positively associated with emotional exhaustion levels (i.e., more expressive suppression is associated with higher levels of emotional exhaustion).

2. To what extent is teachers’ implicit attitude toward emotion regulation moderating the effect of their typical use of emotion regulation strategies on emotional exhaustion?

We expected that teachers who implicitly preferred emotion regulation above expressing emotions would experience lower costs of using effortful emotion regulation strategies, and thus the negative association between using cognitive reappraisal and feelings of emotional exhaustion would be accelerated (i.e., more negative), while the positive association between expressive suppression and emotional exhaustion would be attenuated (i.e., less positive).

In the final step of our analyses, we included contextual and personal factors that have been associated with teacher burnout in previous studies, namely, the quality of the teacher–student relationship, teaching experience, and gender. We wanted to check whether our findings for the first two research questions would change when these variables were entered to the regression model. The teacher–student relationship has often been postulated as an important factor for teacher emotions and well-being (Hargreaves, 2000; Chang, 2009, 2013; Spilt et al., 2011; Van Droogenbroeck et al., 2014). Especially teachers who are both warm (high in communion) and demanding (high in agency) might be less vulnerable for emotional exhaustion (Wubbels et al., 2006; Bondy et al., 2013), but even teachers who have overall favorable and positive relationships with their students may sometimes report negative emotions and burnout symptoms (Donker et al., 2018). Research suggests that also teaching experience needs to be taken into account to understand teachers’ feelings of emotional exhaustion (Grandey, 2000; Troy et al., 2013). It has been found that beginning teachers may experience more intense emotions (Chang, 2009), which in turn may lead to stronger feelings of emotional exhaustion (Harmsen et al., 2018) and attrition of beginning teachers (Buchanan et al., 2013). Furthermore, gender differences have been found both in the use of emotion regulation strategies as well as in the consequences of engaging in emotion regulation. Women are more likely to report the use of emotion regulation strategies in general (Nolen-Hoeksema and Aldao, 2011), but men engage more often in emotion suppression in particular (Grandey, 2000; Spaapen et al., 2014). This could be problematic as using emotion expression has been associated with emotional exhaustion (Moore et al., 2008; Jiang et al., 2016). However, Johnson and Spector (2007) found that females experienced more negative consequences of expressive suppression than men. Although not all studies found gender differences in self-reported cognitive reappraisal (see, for example, Zlomke and Hahn, 2010), there is some evidence for neural differences between men and women engaging in emotion regulation that are potentially related to their implicit attitudes toward emotion regulation (McRae et al., 2008). Given these findings, we also included teacher gender in the present study. Our third research question thus was:

3. To what extent do the quality of the teacher–student relationship, teaching experience, and gender explain additional variance in teachers’ emotional exhaustion?

## Materials and Methods

### Participants

Participants were 94 teachers aged from 20 to 64 years (*M* = 35.26, *SD* = 12.53), and 52.1% of the teachers had less than 5 years of teaching experience. The gender distribution was about equal (55.3% female), and 84% of the teachers was right-handed. The sample consisted of teachers from secondary education (*N* = 49), vocational education (*N* = 36), and a teacher training program for secondary education (*N* = 9). As expected, the groups differed in age [*F*(2,91) = 4.80, *p* = 0.010] and teaching experience [χ^2^(2) = 24.58, *p* < 0.001; Table 1] with vocational education teachers being older than secondary education teachers, and there was a higher percentage of teachers with less than 5 years of teaching experience in the group of secondary education teachers and student teachers. Because teaching experience and age were highly correlated (*r* = 0.75), we included only teaching experience in our analyses. There were no significant differences between the groups in terms of gender [χ^2^(2) = 3.10, *p* = 0.212]. Per teacher, one class of students provided ratings of the teacher–student relationship, which were averaged per teacher. There were on average 19 students per class who gave ratings (*SD* = 6). Most students were aged between 14 and 20.

**TABLE 1:** Teacher characteristics for the full sample and the three separate groups.

		Age (years)			Teaching experience (% < 5 years)	Gender (% female)
	*N*	*M*	*SD*	Range		
Full sample	94	35.26	12.53	20–64	52.1	55.3
Secondary education	49	32.88	12.54	20–63	65.3	49.0
Vocational education	36	39.97	12.05	24–64	22.2	66.7
Student teachers	9	29.33	8.44	23–48	100.0	44.4

### Design and Procedure

The design of the study got ethical approval before data were collected (FETC16-110). Participating teachers and students were informed *a priori* about the research by means of an information letter and were asked to sign an informed consent form. Teachers first completed a computer task to measure their implicit attitude toward emotion regulation versus emotion expression [i.e., the Emotion Regulation-IAT (ER-IAT)] and subsequently completed a digital questionnaire measuring their typical use of emotion regulation strategies and their emotional exhaustion to prevent possible effects of the questionnaires on the ER-IAT. Completing the ER-IAT and questionnaires took a maximum of 30 min per teacher. Students filled in the questionnaire on their perception of the teacher–student relationship during one of their lessons (approximately 5 min).

### Measures

#### Emotional Exhaustion

Emotional exhaustion was measured using a Dutch translation of the MBI (Maslach et al., 1996) with a specific focus on the teaching context [i.e., the Utrecht Burnout Scale for Teachers (UBOS-L); Schaufeli and Van Dierendonck, 2000]. The UBOS-L was used to measure all three aspects of teachers’ burnout: *emotional exhaustion*, *depersonalization*, and *personal accomplishment.* For this study, we only used data from the emotional exhaustion scale as this is often considered to be the most central aspect of burnout (Chang, 2009; Goetz et al., 2015; Arens and Morin, 2016). Emotional exhaustion was measured with eight items, measuring the extent to which a teacher feels “empty” or exhausted due to work-related efforts (e.g., “I feel like I am at the end of my rope”). Items were answered on a seven-point Likert scale bounded by 0 “never” and 6 “daily.” Internal consistency of this scale in the present study was good (α = 0.88).

#### Emotion Regulation Strategies

Teachers’ typical use of emotion regulation strategies was measured using the Dutch version of the Emotion Regulation Questionnaire (ERQ; Gross and John, 2003; Koole and Jostmann, 2004). The ERQ covers the two most well-known emotion regulation strategies *cognitive reappraisal* and *expressive suppression.* Cognitive reappraisal was measured with six items, such as “I control my emotions by changing the way I think about the situation I’m in.” Expressive suppression was measured with four items, for example, “I keep my emotions to myself.” All items were measured on a seven-point Likert scale ranging from 1 “strongly disagree” to 7 “strongly agree.” In the current study, internal consistency was good for the cognitive reappraisal (α = 0.84) and sufficient for the expressive suppression scale (α = 0.73; cf. Koole and Jostmann, 2004).

#### Implicit Attitude Toward Emotion Regulation

Implicit Association Tests were introduced by Greenwald et al. (1998) and have been widely used since then in the field of social psychology and neighboring fields. IATs were developed to measure the strength of automatic associations between categories and aim to test people’s relative preference for one construct over the other. The underlying assumption is that it will take participants longer to categorize words when the combined constructs are not in line with their implicit attitudes. Mauss et al. (2006) developed the ER-IAT to measure individuals’ implicit positive versus negative evaluation of emotion regulation versus emotion expression. The ER-IAT was translated into Dutch for the current study (see Supplementary Material A for a description of the translation process).

The ER-IAT is a computer-based reaction time task in which participants need to assign *target-concept* words such as “controlled” to either the “emotion regulation” or “emotion expression” category and *attribute* words such as “pleasant” to the “positive” or “negative” category. An overview of the words that we used in the present study is included in Supplementary Material A. The words appear one by one in the middle of the screen, and participants assign the word to the category on the top left by pressing a key on the left side of the keyboard (i.e., “d”) or to the category on the right by pressing a key on the right side (i.e., “k”; see Table 2 for screenshots). Participants were asked to respond as quickly as possible, but without making errors. When making an error, participants need to repeat the assignment and attribute the word to the correct category (i.e., built-in error penalty).

**TABLE 2:** Overview of the different task blocks in the Emotion Regulation–Implicit Association Test (ER-IAT).

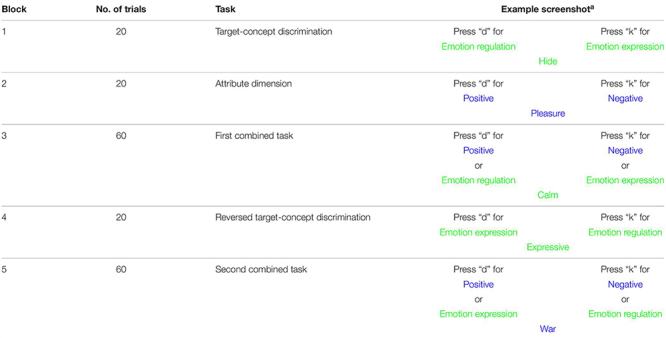

*^*a*^Green color refers to target-concept words. Blue color refers to attribute words.*

The ER-IAT consists of five blocks, of which Blocks 1, 2, and 4 are practice trials (20 trials per block; see Table 2 for an overview of the blocks). In Block 1, the task starts with an introduction of the target-concept discrimination by assigning words to one of the following categories: (a) emotion regulation or (b) emotion expression. In Block 2, the attribute dimension is introduced with two categories: (a) positive or (b) negative. In Block 3, the previous two blocks were superimposed. Participants were asked to assign words to one of two combined categories: (a) emotion regulation or positive on the left and (b) emotion expression or negative on the right side of the screen. This block consists of 20 practice trials and 40 test trails. In Block 4, the participant learned to reverse the response assignment for the target discrimination of Block 1 (i.e., the target-concept category on the left and right switch places; Table 2). In Block 5, participants were again asked to categorize items into two combined categories. However, the response assignment for the target discrimination is still reversed. Thus, participants need to assign words to (a) emotion expression or positive and (b) emotion regulation or negative. Similar to Block 3, this block consisted of 20 practice trials and 40 test trails. The order of the blocks was not counterbalanced, as we were interested in the relative size of the ER-IAT effect across participants (in line with Mauss et al., 2006).

We followed the algorithm of Greenwald et al. (2003) to score the ER-IAT reaction time data (*D*) using both practice and test trials of Blocks 3 and 5. To account for individual variability of the latencies, average latencies of the trials were divided by an individual’s standard deviation. The final ER-IAT score was calculated by subtracting averages of Block 3 from averages of Block 5. The stronger (or weaker) the association of particular pairs of categories (e.g., “emotion regulation” and “positive”) is, the lower (or higher) the response times are compared to other category pairs (e.g., “emotion expression” and “positive”). A score above zero indicated an implicit positive attitude toward emotion regulation, and a score below zero implied an implicit positive attitude toward emotion expression. Good internal consistency was found using split-half methodology over Blocks 3 and 5 (α = 0.80).

### Interpersonal Teacher–Student Relationship

The Questionnaire on Teacher Interaction (QTI; Wubbels et al., 1985) was used to chart student perceptions of the teacher–student relationship in terms of teachers’ *Agency* and *Communion* in class. Agency refers to taking the lead, social influence, or dominance. Communion refers to friendliness, affection, or warmth. Twenty-four items were used to measure both dimensions and as is customary in circumplex measures, each item was weighted separately for each interpersonal dimension. For example, “this teacher is strict” was weighted strongly positive for Agency (i.e., 0.92) and less strongly and negative for teacher Communion (i.e., -0.38). The item “this teacher is patient” on the other hand was weighted moderately negative (i.e., -0.38) for Agency and strongly positive for Communion (i.e., 0.92; see for a comprehensive explanation Den Brok et al., 2004). Items were answered on a five-point Likert scale bounded by 1 “(almost) never” and 5 “(almost) always.” Students’ scores on the items were averaged at the class level. In the current study, we found good internal consistency for both agency and communion at the class level, with α = 0.89 and α = 0.93, respectively (cf. Den Brok et al., 2004).

### Data Analysis

Hierarchical multiple regression analyses were used to examine the effect of teachers’ typical use of and implicit attitudes toward emotion regulation strategies on teachers’ level of emotional exhaustion (step 1) and to investigate the moderating role of implicit attitudes toward emotion regulation (step 2). In a third step, we tested the effect of including our covariates. The statistical analyses were carried out using SPSS version 24. Based on visual inspection of scatterplots, linearity could be assumed. Variables were normally distributed (skewness and kurtosis values were between -1.96 and 1.96 for all variables). To avoid problems with multicollinearity, the interaction terms of typical use of and implicit attitudes toward emotion regulation were created by multiplying the centered scores (Helm and Mark, 2012). The assumption of homoscedasticity was met, and residuals were independent and normally distributed. Furthermore, one univariate outlier was identified for the interaction term between expression suppression and the ER-IAT (z-score = 3.79), but further inspection showed that there was no reason to delete this value. There were no multivariate outliers (Cook’s distance < 1 for all variables).

We investigated the research questions in separate models for cognitive reappraisal (model 1) and expressive suppression (model 2) to increase the power. The steps are described below for model 1, but were similar for model 2 (see Table 3 for an overview of the regression models). For model 1a, cognitive reappraisal and implicit attitudes toward emotion regulation were entered in the first step as predictors of emotional exhaustion. In the second step, to explore the added value of including the moderating role of implicit attitudes toward emotion regulation, the interaction effect between cognitive reappraisal and implicit attitudes was added to the model. Graphical representations were made to interpret the direction of the moderation (Helm and Mark, 2012) using PROCESS version 3.3 in SPSS version 24. In the third step, teaching experience and gender were added to the model as potential covariates. These were included only in the final step because of the limited sample size and in order to assess the robustness of the models tested in steps 1 and 2. A separate model (model 1b) was estimated where we added teacher agency and communion in the third step, since data on the teacher–student relationship were only available for a subsample (*N* = 72). Because all missing data for the teacher–student relationship were located in the group of teachers with less than 5 years of experience (secondary education teachers *N* = 21 and student teachers *N* = 1) and thus not missing completely at random (MCAR), we compared a model using the default option listwise deletion (LD, *N* = 72) and using multiple imputation (MI) with five imputations (*N* = 94). All predictor and outcome variables and several auxiliary variables (i.e., handedness, age, and education type) were included as predictors for the imputations (Collins et al., 2001).

**TABLE 3:** Overview of conducted hierarchical multiple regression models.

Model	*N*	Step 1	Step 2	Step 3
1a	94	CR, ER-IAT	CR*ER-IAT	Teaching experience, gender
1b (LD)	72	CR, ER-IAT	CR*ER-IAT	Agency, communion
1b (MI)	94	CR, ER-IAT	CR*ER-IAT	Agency, communion
2a	94	ES, ER-IAT	ES*ER-IAT	Teaching experience, gender
2b (LD)	72	ES, ER-IAT	ES*ER-IAT	Agency, communion
2b (MI)	94	ES, ER-IAT	ES*ER-IAT	Agency, communion

*LI, listwise deletion; MI, multiple imputation; CR, cognitive reappraisal; ES, expressive suppression; ER-IAT, Emotion Regulation-Implicit Association Test.*

## Results

### Descriptive Statistics

Descriptive statistics are presented in Table 4. On average, teachers scored low on emotional exhaustion (*M* = 1.64, *SD* = 1.05), but scores ranged from 0 (never) to 5.13 (several times a week to daily). They reported significantly more use of cognitive reappraisal (*M* = 4.66, *SD* = 1.01) than expression suppression [*M* = 3.30, *SD* = 1.09; *t*(93) = 9.487, *p* < 0.001]. Teachers’ implicit attitude toward emotion regulation versus emotion expression ranged from negative to positive with a mean of −0.09 (*SD* = 0.45), indicating on average a slight preference for emotion expression compared to emotion regulation. Teachers’ communion levels were moderate and teachers’ agency levels were relatively lower than in prior research (Claessens et al., 2016), which can be explained by the relatively large number of teachers with less than 5 years of experience in our sample (cf. Brekelmans et al., 2005). Indeed, we found a significant association between teaching experience and agency (*r* = 0.33; Table 5).

**TABLE 4:** Descriptive statistics for the full sample and the three separate groups.

	Full sample				Secondary education				Vocational education				Student teachers			
	*N*	*M*	*SD*	Range	*N*	*M*	*SD*	Range	*N*	*M*	*SD*	Range	*N*	*M*	*SD*	Range
EE	94	1.64	1.05	0 – 5.13	49	1.73	1.09	0 – 5.13	36	1.34	0.84	0 – 3.88	9	2.43	1.16	0.75 – 4.13
CR	94	4.66	1.01	2.00 – 7.00	49	4.61	1.03	2.00 – 6.67	36	4.94	0.91	2.83 – 7.00	9	3.77	0.89	2.83 – 5.33
ES	94	3.30	1.09	1.25 – 5.50	49	3.44	1.13	1.25 – 5.50	36	3.19	0.96	1.25 – 4.75	9	2.97	1.32	1.25 – 4.75
ER-IAT	94	−0.09	0.46	−1.12 – 0.96	49	−0.09	0.48	−0.89 – 0.96	36	−0.08	0.41	−0.90 – 0.61	9	−0.10	0.57	−1.12 – 0.47
Ag	72	0.21	0.11	−0.07 – 0.45	28	0.22	0.09	−0.07 – 0.34	36	0.23	0.11	0.00 – 0.45	8	0.07	0.11	−0.07 – 0.27
Com	72	0.45	0.17	0.02 – 0.76	28	0.46	0.17	0.02 – 0.72	36	0.46	0.18	0.03 – 0.76	8	0.42	0.10	0.24 – 0.54

*EE, emotional exhaustion; CR, cognitive reappraisal; ES, expressive suppression; ER-IAT, Emotion Regulation – Implicit Association Test; Ag, Agency; Com, Communion.*

**TABLE 5:** Pearson correlations for the full sample (*N* = 94).

Variable	1	2	3	4	5	6	7	8
1 Emotional exhaustion	–							
2 Cognitive reappraisal	−0.24*	–						
3 Expressive suppression	–0.06	0.14	–					
4 Implicit attitudes	0.01	–0.02	0.21*	–				
5 Agency^a^	−0.31**	0.10	–0.00	0.09	–			
6 Communion^a^	–0.05	0.02	0.26*	–0.13	–0.03	–		
7 Experience^b^	−0.23*	0.07	–0.11	–0.01	0.33**	0.04	–	
8 Gender^c^	0.03	0.27**	–0.14	–0.20	–0.07	–0.07	0.05	–

*p < 0.05; **p < 0.01. ^*a*^*N* = 72. ^*b*^0 = less than 5 years, 1 = more than 5 years. ^*c*^0 = male, 1 = female.

### Correlational Analyses

The correlational analyses (Table 5) showed that teachers who reported higher levels of emotional exhaustion were more likely to report less use of cognitive reappraisal (*r* = −0.24), were perceived as lower on teacher agency by students (*r* = −0.31), and had less teaching experience (*r* = −0.23). Although there was no correlation between teachers’ implicit attitude toward emotion regulation and their feelings of emotional exhaustion, we found that teachers who had an implicit positive attitude toward emotion regulation (as opposed to emotion expression) tended to report somewhat more use of expressive suppression strategies (*r* = 0.21). Female teachers reported more use of cognitive reappraisal emotion regulation strategies (*r* = 0.27). Finally, we found that students tended to report higher levels of communion in classrooms of teachers who reported to use more expressive suppression (*r* = 0.26).

### Hierarchical Multiple Regression Analyses

#### Cognitive Reappraisal

To examine the relationship between cognitive reappraisal and emotional exhaustion, a hierarchical multiple regression analysis was conducted. Table 6 presents a summary of the results, including unstandardized regression coefficients (*b*), standardized regression coefficients (β), and the standard errors (*SE*) of the unstandardized regression coefficients. In step 1, we found that cognitive reappraisal was a significant predictor of emotional exhaustion (β = −0.24, *p* = 0.019) meaning that teachers who reported more use of cognitive reappraisal scored lower on emotional exhaustion and vice versa. Cognitive reappraisal and implicit attitudes toward emotion regulation accounted for a non-significant 5.9% of the variance in emotional exhaustion. Adding the interaction term between cognitive reappraisal and implicit attitudes toward emotion regulation in step 2 explained an additional non-significant 1.3% of the variance in emotional exhaustion. Cognitive reappraisal remained a significant predictor (β = −0.25, *p* = 0.015), but implicit attitudes toward emotion regulation were not a statistically significant moderator (see Figure B1 in Supplementary Material B for a visualization). In the third step, we included teaching experience and gender, and together these factors explained a significant 12.4% of the variance in emotional exhaustion. Next to cognitive reappraisal, teaching experience was a significant predictor of emotional exhaustion. Teachers with less than 5 years of experience scored on average 0.44 higher on emotional exhaustion than teachers with more than 5 years of experience.

**TABLE 6:** Results of the hierarchical multiple regression for Model 1a.

Step		*b*	*SE*	β	*t*	*p*	Δ*R*^2^	*df*	Δ*F*	*p*
1							0.06	2, 91	2.87	0.062
	CR	−0.25	0.12	−0.24	−2.39	0.019				
	ER−IAT	0.02	0.23	0.01	0.09	0.931				
2							0.01	1, 90	1.23	0.271
	CR	−0.26	0.11	−0.25	−2.48	0.015				
	ER−IAT	0.02	0.23	0.01	0.10	0.922				
	CR*ER−IAT	0.25	0.23	0.11	1.11	0.271				
3							0.05	2, 88	2.62	0.079
	CR	−0.27	0.11	−0.26	−2.53	0.013				
	ER−IAT	0.06	0.23	0.03	0.27	0.786				
	CR*ER−IAT	0.23	0.23	0.10	1.02	0.309				
	Gender^a^	0.21	0.22	0.10	0.92	0.360				
	Experience^b^	−0.44	0.21	−0.21	−2.12	0.037				

*CR, cognitive reappraisal; ER-IAT, Emotion Regulation-Implicit Association Test. ^*a*^0 = male, 1 = female. ^*b*^0 = less than 5 years, 1 = more than 5 years.*

To explore the effect of the teacher–student relationship on teachers’ emotional exhaustion, we included agency and communion in the third step for a subsample of teachers (*N* = 72; see bold coefficients in Table 7). This model explained a significant 19.2% in the variance of emotional exhaustion. Both cognitive reappraisal (β = −0.28, *p* = 0.017) and agency (β = −0.32, *p* = 0.007) were significant predictors of emotional exhaustion. To test the effect of the teacher–student relationship for the whole sample (*N* = 94), we conducted MI with five imputations. Table 7 presents the pooled results of the regression analysis in italic. Percentages of explained variance and standardized regression coefficients cannot be computed for pooled data. Similar to the models above, cognitive reappraisal (*b* = −0.22, *p* = 0.028) and teacher agency (*b* = −2.85, *p* = 0.006) predicted teachers’ emotional exhaustion significantly.

**TABLE 7:** Results of the hierarchical multiple regressions for model 1b using LD and MI.

		*b*		*SE*		β	*t*		*p*		Δ*R*^2^	*df*	Δ*F*	*p*
									
Step		LD	*MI*	LD	*MI*	LD	LD	*MI*	LD	*MI*	LD	LD	LD	LD
1											**0.09**	**2, 69**	**3.42**	**0.038**
	CR	−**0.28**	−*0.25*	**0.11**	*0.11*	−**0.29**	−**2.52**	−*2.39*	**0.014**	*0.017*				
	ER-IAT	**0.09**	*0.02*	**0.24**	*0.23*	**0.04**	**0.38**	*0.09*	**0.706**	*0.931*				
2											**0.01**	**1, 68**	**0.42**	**0.519**
	CR	−**0.29**	−*0.26*	**0.11**	*0.11*	−**0.30**	−**2.58**	−*2.49*	**0.012**	*0.013*				
	ER-IAT	**0.08**	*0.03*	**0.24**	*0.23*	**0.04**	**0.34**	*0.14*	**0.733**	*0.889*				
	CR*ER-IAT	**0.15**	*0.25*	**0.24**	*0.23*	**0.08**	**0.65**	*1.11*	**0.519**	*0.268*				
3											**0.10**	**2, 66**	**3.91**	**0.025**
	CR	−**0.26**	−*0.22*	**0.11**	*0.10*	−**0.28**	−**2.45**	−*2.20*	**0.017**	*0.028*				
	ER-IAT	**0.13**	*0.10*	**0.23**	*0.22*	**0.06**	**0.55**	*0.45*	**0.583**	*0.651*				
	CR*ER-IAT	**0.28**	*0.29*	**0.23**	*0.22*	**0.14**	**1.21**	*1.34*	**0.231**	*0.181*				
	Agency	−**2.56**	−*2.85*	**0.93**	*1.01*	−**0.32**	−**2.77**	−*2.81*	**0.007**	*0.006*				
	Communion	−**0.31**	−*0.90*	**0.63**	*0.75*	−**0.06**	−**0.49**	−*1.19*	**0.623**	*0.242*				

*Bold coefficients refer to results from the analyses using listwise deletion. Italic coefficients refer to results from using multiple imputation. Standardized coefficients and change statistics are not available when using multiple imputation. LD, listwise deletion; MI, multiple imputation; CR, cognitive reappraisal; ER-IAT, Emotion Regulation-Implicit Association Test.*

#### Expressive Suppression

Table 8 presents the results for teachers’ use of expressive suppression (model 2a). Expressive suppression and implicit attitudes toward emotion regulation accounted for a negligible 0.5% of variance in emotional exhaustion; neither expressive suppression nor implicit attitudes toward emotion regulation were significant predictors. The model including also the interaction term between expressive suppression and implicit attitudes toward emotion regulation explained a non-significant 1.4% of the variance in emotional exhaustion. Implicit attitudes toward emotion regulation were not a statistically significant moderator of the relationship between expressive suppression and emotional exhaustion (see Figure B2 in Supplementary Material B for a visualization). Including teaching experience and gender in the third step led to an extra 5.5% explained variance of emotional exhaustion. We found that teachers with less than 5 years of experience scored on average 0.49 higher on emotional exhaustion than teachers with more than 5 years of experience.

**TABLE 8:** Results of the hierarchical multiple regression for Model 2a.

Step		*b*	*SE*	β	*t*	*p*	Δ*R*^2^	*df*	Δ*F*	*p*
1							0.01	2, 91	0.22	0.805
	ES	−0.07	0.10	−0.07	−0.65	0.518				
	ER-IAT	0.06	0.24	0.03	0.25	0.804				
2							0.01	1, 90	0.85	0.360
	ES	−0.06	0.10	−0.07	−0.61	0.542				
	ER-IAT	0.02	0.25	0.01	0.07	0.945				
	ES*ER-IAT	−0.20	0.21	−0.10	−0.92	0.360				
3							0.06	2, 88	2.58	0.082
	ES	−0.08	0.10	−0.09	−0.82	0.413				
	ER-IAT	0.04	0.25	0.02	0.17	0.863				
	ES*ER-IAT	−0.19	0.21	−0.09	−0.89	0.378				
	Gender^a^	0.08	0.22	0.04	0.35	0.726				
	Experience^b^	−0.49	0.22	−0.23	−2.25	0.027				

*ES, expressive suppression; ER-IAT, Emotion Regulation-Implicit Association Test. ^*a*^0 = male, 1 = female. ^*b*^0 = less than 5 years, 1 = more than 5 years.*

**FIGURE 1:**
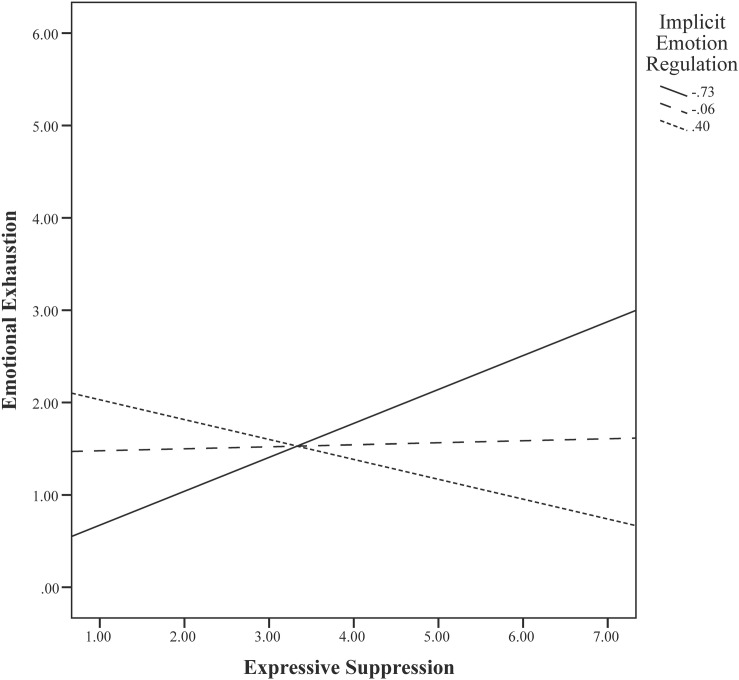
Visualization of the moderating effect of implicit attitudes toward emotion regulation on the relationship between expressive suppression and emotional exhaustion within the subsample (step 2).

Table 9 presents a summary of the results for model 2b including teacher agency and communion. Using LD (*N* = 72), we found that a significant 15.8% of the variance of emotional exhaustion could be explained by our predictors. In contrast to the previous models, teachers’ implicit attitude toward emotion regulation was a significant moderator of the relationship between expressive suppression and emotional exhaustion in step 2 (β = −0.27, *p* = 0.030). Figure 1 illustrates that teachers who showed a stronger implicit positive attitude toward emotion regulation and reported making more use of expressive suppression strategies reported lower emotional exhaustion. On the other hand, teachers who showed a stronger implicit positive attitude toward emotion expression and reported making more use of expressive suppression strategies reported higher levels of emotional exhaustion. A potential explanation for this finding might be that this subsample contained less teachers with limited years of experience (<5 years) than the full sample. Since we found that teaching experience was a significant predictor of emotional exhaustion in model 1/2a, it is possible that the predictive value of the interaction between using expressive suppression and implicit attitudes toward emotion regulation (as opposed to emotion expression) only holds for teachers with more years of experience. The moderation effect failed to reach significance in the final step (β = −0.23, *p* = 0.055), where we found agency to be the only significant predictor of emotional exhaustion (β = −0.29, *p* = 0.014). In our model using MI (*N* = 94), implicit attitudes toward emotion regulation did not significantly moderate the relationship between expressive suppression and emotional exhaustion (*b* = −0.20, *p* = 0.326). Again, only agency predicted emotional exhaustion significantly (*b* = −3.01, *p* = 0.005).

**TABLE 9:** Results of the hierarchical multiple regression for Model 2b using LD and MI.

		*b*		*SE*		β	*t*		*p*		Δ*R*^2^	*df*	Δ*F*	*p*
									
Step		LD	*MI*	LD	*MI*	LD	LD	*MI*	LD	*MI*	LD	LD	LD	LD
1											**0.01**	**2, 69**	**0.23**	**0.794**
	ES	**0.01**	−*0.07*	**0.10**	*0.10*	**0.02**	**0.14**	−*0.65*	**0.892**	*0.517*				
	ER-IAT	**0.16**	*0.06*	**0.25**	*0.24*	**0.08**	**0.65**	*0.25*	**0.517**	*0.803*				
2											**0.07**	**1, 68**	**4.90**	**0.030**
	ES	**0.04**	−*0.06*	**0.10**	*0.10*	**0.04**	**0.34**	−*0.60*	**0.732**	*0.550*				
	ER-IAT	**0.03**	*0.01*	**0.25**	*0.25*	**0.01**	**0.12**	*0.05*	**0.909**	*0.963*				
	ES*ER-IAT	−**0.51**	−*0.20*	**0.23**	*0.21*	−**0.27**	−**2.21**	−*0.92*	**0.030**	*0.357*				
3											**0.08**	**2, 66**	**3.30**	**0.043**
	ES	**0.04**	−*0.03*	**0.10**	*0.11*	**0.05**	**0.41**	−*0.29*	**0.680**	*0.770*				
	ER-IAT	**0.09**	*0.07*	**0.25**	*0.24*	**0.04**	**0.35**	*0.29*	**0.731**	*0.775*				
	ES*ER-IAT	−**0.44**	−*0.20*	**0.23**	*0.20*	−**0.23**	−**1.95**	−*0.98*	**0.055**	*0.326*				
	Agency	−**2.36**	−*3.01*	**0.93**	*1.04*	−**0.29**	−**2.54**	−*2.89*	**0.014**	*0.005*				
	Communion	−**0.30**	−*0.80*	**0.67**	*0.87*	−**0.05**	−**0.45**	−*0.92*	**0.653**	*0.369*				

*Bold coefficients refer to results from the analyses using listwise deletion. Italic coefficients refer to results from using multiple imputation. Standardized coefficients and change statistics are not available when using multiple imputation. LD, listwise deletion; MI, multiple imputation; ES, expressive suppression; ER-IAT, Emotion Regulation-Implicit Association Test.*

## Discussion

The present study investigated the association of teachers’ typical use of emotion regulation strategies (i.e., cognitive reappraisal and expressive suppression) with teachers’ feelings of emotional exhaustion and explored the moderating effect of implicit attitudes toward emotion regulation versus emotion expression on this association with an IAT. We included covariates that have been associated with teachers’ burnout level in previous studies (i.e., the teacher–student relationship, teaching experience, and gender). This study marks one of the first attempts to examine the potential moderating role of teachers’ implicit preferences on the relation between their typical use of emotion regulation strategies and their emotional exhaustion.

### Emotion Regulation Strategies and Emotional Exhaustion

In line with our hypothesis, we found that teachers who reported more use of cognitive reappraisal tended to report less emotional exhaustion. The strength of this association was comparable to previous studies in the teaching context (Tsouloupas et al., 2010; Andela and Truchot, 2015). This suggests that cognitive reappraisal is an adaptive emotion regulation strategy also in the teaching context (e.g., Gross and John, 2003; Barber et al., 2011; Becker et al., 2015). It might be worthwhile for teacher training and professional development programs for teachers to develop a training program focused on practicing cognitive reappraisal. Recent studies in other fields have shown positive effects of such trainings on well-being and stress reduction (Denny and Ochsner, 2014; Ranney et al., 2017).

In contrast to findings from other studies (mainly outside the educational context), we did not find a significant positive relationship between expressive suppression and emotional exhaustion. A possible explanation might be that expressive suppression can sometimes be functional for teachers in the classroom context because it conforms to display rules (cf. Frenzel, 2014). Sutton et al. (2009), for example, differentiated between regulating positive and negative emotions and found that teachers disagreed on the effectiveness of expressing negative emotions. Two-thirds of the teachers in their study reported less teaching effectiveness after expressing negative emotions (Sutton et al., 2009) and thus seemed to have a preference for suppressing their negative emotions during teaching.

### Moderating Effect of Implicit Attitudes Toward Emotion Regulation

Contrary to our hypothesis, implicit positive attitudes toward emotion regulation (as opposed to emotion expression) did not directly predict emotional exhaustion levels nor did it moderate the relationship between teachers’ typical use of emotion regulation strategies and their feelings of emotional exhaustion in the full sample. This contradicts previous findings by Hopp et al. (2011) outside the educational context, who found a positive moderating effect of an implicit positive attitude toward emotion regulation when combined with the use of cognitive reappraisal strategies. The absence of this moderating effect in our sample of teachers is possibly due to relatively strong display rules in the educational setting, which might diminish the effect of teachers’ individual preferences or implicit attitudes (Gosserand and Diefendorff, 2005; Mauss et al., 2008).

Interestingly, we did find a significant moderation effect of implicit attitudes toward emotion regulation in a subsample with more experienced teachers. More experienced teachers who demonstrated an implicit positive attitude toward regulating their emotions and who reported making more use of expressive suppression strategies tended to report lower levels of emotional exhaustion. Likewise, teachers who showed an implicit positive attitude toward emotion expression and reported making more use of expressive suppression strategies reported somewhat higher levels of emotional exhaustion. The effect of teachers’ typical use of and implicit attitudes toward emotion regulation versus emotion expression on their emotional exhaustion might thus be dissimilar for teachers with different levels of experience. We found that not only the levels of emotional exhaustion were highest for teachers with less than 5 years of experience, but they also used less cognitive reappraisal compared to more experienced teachers. Sutton and Harper (2009) suggest that beginning teachers might experience high stress levels, which may result in more difficulty in regulating their emotions and therefore, they may directly express their emotions more often. Experienced teachers have been found to be more likely to regulate in the immediate situation by either reappraising or suppressing the emotion (Sutton and Harper, 2009), and for them it might be more important to have an implicit positive attitude toward emotion regulation (as opposed to emotion expression) as a buffer against the potential negative effects of expressive suppression. Experienced teachers might encounter negative consequences of expressive suppression only when they have a preference for emotion expression. Along similar lines, for teachers who have internalized the display rules of emotion regulation, using expressive suppression may not be harmful. It should be noted that the moderation effect was not significant anymore when teacher agency was included in the model, thus the higher agency level of more experienced teachers might compensate for the potential negative effects of using expressive suppression while having an implicit positive attitude toward emotion expression.

### Contextual and Personal Factors

We found that teachers with higher interpersonal agency levels and more teaching experience reported less emotional exhaustion. This is in line with the finding that the strategy–situation fit is an important protective factor against developing burnout symptoms (Troy et al., 2013; Haines et al., 2016). Furthermore, it supports earlier findings that less experienced teachers report more tension and negative emotions (Harmsen et al., 2018). This is important, as emotional exhaustion is a major reason for beginning teachers to quit the profession (Buchanan et al., 2013). Hence, the first 5 years of teaching may serve as a sensitive period for promoting regulation strategies that help to lower feelings of stress and emotional exhaustion. Teachers’ level of communion and teachers’ gender were not associated with their level of emotional exhaustion. However, we found that teachers who were perceived as being relatively high on communion reported more use of expressive suppression strategies, which may indicate that expressive suppression could help to build more positive relationships with students. Further, female teachers reported more use of cognitive reappraisal strategies than men, which is in line with previous findings (Nolen-Hoeksema and Aldao, 2011). Research is needed to further examine the potential indirect effects of these covariates on teachers’ emotional exhaustion *via* differential use of emotion regulation strategies.

### Limitations and Future Directions

The present study was one of the first to test the role of teachers’ typical use of emotion regulation strategies and their implicit attitudes toward emotion regulation in teachers’ level of emotional exhaustion. The findings of the current study should be interpreted with care, and replication studies are needed to validate the results. In future research, it should be tested whether the differential role of an implicit preference for emotion regulation versus emotion expression for beginning versus more experienced teachers holds in a larger sample. Other aspects of teacher burnout—such as depersonalization or personal accomplishment—should be investigated to see if their association with emotion regulation differs from the findings with regard to emotional exhaustion. Also, future studies could integrate more diverse implicit measures of emotion regulation, such as physiological measures (e.g., Donker et al., 2018) or student ratings of teachers’ emotion regulation (e.g., Jiang et al., 2016) to get a more integrative view of implicit processes in teachers’ emotion regulation.

Although the effects were on average small, they were similar to other studies on teacher burnout (e.g., Evers et al., 2004) and are in line with the idea that many factors interact in predicting teachers’ level of burnout (Chang, 2009; Frenzel, 2014). It would be interesting for future research on teacher emotions and burnout to make more use of measures of implicit preferences for emotion regulation versus emotion expression. We found a small positive correlation between teachers’ use of expressive suppression and their implicit attitude toward emotion regulation. This suggests that the ER-IAT might tap into the motivation to use expressive suppression beyond having only a positive attitude toward emotion regulation.

A limitation is that we used student ratings of the teacher–student relationship. Although their reliability and validity have been shown in previous studies (Wubbels and Brekelmans, 2005; Den Brok et al., 2006), what teachers themselves think about the interpersonal relationship with students might be more strongly related to their own well-being (Aldrup et al., 2018). Finally, it should be noted that our findings are correlational, and thus, we cannot draw any causal conclusions. It is possible that more emotionally exhausted teachers might use cognitive reappraisal to a lesser degree because they do not have the cognitive capacity left to engage in emotion regulation (Richards and Gross, 1999).

## Conclusion

Notwithstanding these limitations and suggestions for further research, the present study adds to our knowledge about emotion regulation in teachers. First of all, there were large differences between teachers in how their emotion regulation strategies and preferences were associated with feelings of emotional exhaustion. This illustrates that the potential benefits and costs of emotion regulation may differ among teachers and that there may be potentially important moderating variables. The results replicated previous findings about the benefits of using cognitive reappraisal emotion regulation strategies and the important role of experience and interpersonal agency in reducing teachers’ feelings of emotional exhaustion in general. A moderating effect of implicit attitudes toward emotion regulation on the association between teachers’ typical use of emotion regulation strategies and their emotional exhaustion level was only found in a subsample with more experienced teachers. This suggests that having an implicit positive attitude toward emotion regulation (as opposed to emotion expression) might help to attenuate emotional exhaustion, especially when one is using expressive suppression strategies, which are common in educational settings due to the display rules for teachers. Studies such as these can inform teacher educators on how to better prepare teachers for their emotional job or support them during professional development courses, for instance, by discussing effective emotion regulation strategies while taking into account the role of more implicit attitudes, both in beginning and more experienced teachers.

## Data Availability Statement

The datasets generated for this study are available on request to the corresponding author.

## Ethics Statement

The studies involving human participants were reviewed and approved by the Ethics Review Board of the Faculty of Social and Behavioral Sciences. Written informed consent from the participants’ legal guardian/next of kin was not required to participate in this study in accordance with the national legislation and the institutional requirements.

## Author Contributions

MD, TG, and TM designed the study. MD recruited participants and collected the data. MD and ME analyzed the data and drafted the manuscript. All authors contributed to manuscript revision and read and approved the submitted version.

## Conflict of Interest

The authors declare that the research was conducted in the absence of any commercial or financial relationships that could be construed as a potential conflict of interest.
