# Supplementary material for: Teachers’ Emotional Exhaustion: Associations With Their Typical Use of and Implicit Attitudes Toward Emotion Regulation Strategies
Source: Front Psychol. 2020 May 29;11:867. doi: 10.3389/fpsyg.2020.00867 (PMC7273523; doi:10.3389/fpsyg.2020.00867)
Supplement: Supplementary file 1 [file Data_Sheet_1.pdf]

Donker, M. H., Erisman, M. C., Van Gog, T., & Mainhard, T. (2020). Teachers' emotional exhaustion: Associations with their typical use of and implicit attitude towards emotion regulation strategies. *Frontiers in Psychology, 11:867*. doi:10.3389/fpsyg.2020.00867

Supplementary material A:

Development of the Dutch ER-IAT

For the purpose of the current study, we translated the ER-IAT as developed by Mauss et al. (2006) into Dutch. Our goal was to stay as close as possible to the validated original version, while making sure that the test fitted well to the Dutch language context. See Table A1 for the items in the original IAT and the revised Dutch version. The process of modification is described below.

First, the words of the original 'emotion regulation' and 'emotion expression' categories were translated to Dutch separately by two researchers (the first author and a psychology student) fluent in both Dutch and English with the help of online dictionaries. Translations were discussed until agreement on all items was reached. Second, to prevent artefacts, the chosen stimuli were checked for average word frequency and word length using a word frequency database for Dutch words based on film subtitles containing 43.8 million words (Keuleers, Brysbaert, & New, 2010). On average, familiarity of the emotion regulation items (M = 655.60, SE = 520.42) did not differ significantly from the emotion expression items (M = 163.20, SE = 214.57), $t(8) = -1.95$, $p = .086$. Furthermore, word length of the emotion regulation items did not differ significantly (M = 9.40, SE = 3.51) from the emotion expression items (M = 9.20, SE = 0.84), $t(8) = -.124$, $p = .904$. Third, after the initial translation, synonyms and other words that could be associated with the categories were added.

To decide on the final items, a questionnaire was composed to test for the interpretation of the words. Participants were asked to answer the following questions: 'How positive or negative do you find the following words?' on a scale ranging from 1 'very negative' to 7 'very positive', and 'How well do you think the following words fit emotion regulation or emotion expression?' on a scale from 1 'regulation' to 7 'expression'. The questionnaire was completed by 155 participants from the general population (124 female; $M_{age} = 32$, SD_{age} = 14.3). The age and gender distribution matched the distribution among teachers in our sample. We checked whether the words in the emotion regulation and emotion expression categories were similar in valence and different in their fit to the category. Valence of the emotion regulation words (M = 4.24, SE = 1.34) did not differ significantly from the emotion expression words (M = 4.61, SE = .54), $t = -.627$, $p = .545$. In addition, the items differed on their fit to the categories, with the emotion regulation category being significantly lower (M = 2.29, SE = .43) than emotion expression words (M = 5.77, SE = .44), $t = -13.752$, $p < .001$. Therefore, we concluded that the categories were well matched regarding positivity/negativity and there was a significant distinction between the items of the two categories.

The resulting categories were again checked on average word length and frequency, which revealed no significant difference between the two categories. This led to the final words for emotion regulation and emotion expression used in the current study (see bolded words in Table A1). We used six words for both the emotion regulation and emotion expression category to be able to delete one afterwards if they did not perform well. This was however not necessary and we thus kept all six words in the analyses. For the positive and negative words, we used validated words from a previous Dutch IAT study (Van den Bergh, Denessen, Hornstra, Voeten, & Holland, 2010).

Donker, M. H., Erisman, M. C., Van Gog, T., & Mainhard, T. (2020). Teachers' emotional exhaustion: Associations with their typical use of and implicit attitude towards emotion regulation strategies. *Frontiers in Psychology, 11:867*. doi:10.3389/fpsyg.2020.00867

Table A1

Overview of English Items Used in the Original ER-IAT (Mauss et al., 2006) and Dutch Items Used in the Current Study and their Translation

**Emotion Regulation**		**Emotion Expression**		**Positive**		**Negative**	
**English**	**Dutch**	**English**	**Dutch**	**English**	**Dutch**	**English**	**Dutch**
**Controlled**	**Gecontroleerd**	**Expressive**	**Expressief**	**Pleasant**	**Plezier**	**Negative**	*Negatief*
**Cool**	*Koel*	**Emotional**	**Emotioneel**	**Good**	*Goed*	**Bad**	*Slecht*
**Hide**	**Verbergen**	**Reveal**	**Onthullen**	**Gold**	*Goud*	**Gloom**	*Somber*
**Contain**	**Beheersen**	**Disclose**	*Openbaren*	**Honor**	*Eer*	**Filth**	*Vuil*
**Suppress**	**Onderdrukken**	**Discharge**	*Ontladen*	**Lucky**	**Geluk**	**Rotten**	*Verrot*
Calm	**Kalm**	*Impulsive*	**Impulsief**	*Peace*	**Vrede**	*War*	**Oorlog**
Cautious	**Bedachtzaam**	*Express*	**Uiten**	*Paradise*	**Paradijs**	*Hell*	**Hel**
		Agitated	**Opgewonden**	*Success*	**Succes**	*Pain*	**Pijn**
						Anxiety	**Angst**
						Disaster	**Ramp**

Note. English words in bold were used in the original ER-IAT and Dutch words in bold were used in the current study.
