# Supplementary material for: Teachers’ Emotional Exhaustion: Associations With Their Typical Use of and Implicit Attitudes Toward Emotion Regulation Strategies
Source: Front Psychol. 2020 May 29;11:867. doi: 10.3389/fpsyg.2020.00867 (PMC7273523; doi:10.3389/fpsyg.2020.00867)
Supplement: Supplementary file 2 [file Data_Sheet_2.pdf]

Donker, M. H., Erisman, M. C., Van Gog, T., & Mainhard, T. (2020). Teachers' emotional exhaustion: Associations with their typical use of and implicit attitude towards emotion regulation strategies. *Frontiers in Psychology, 11:867*. doi:10.3389/fpsyg.2020.00867

**Supplementary material B:**

**Graphical representations of the insignificant moderation effects**

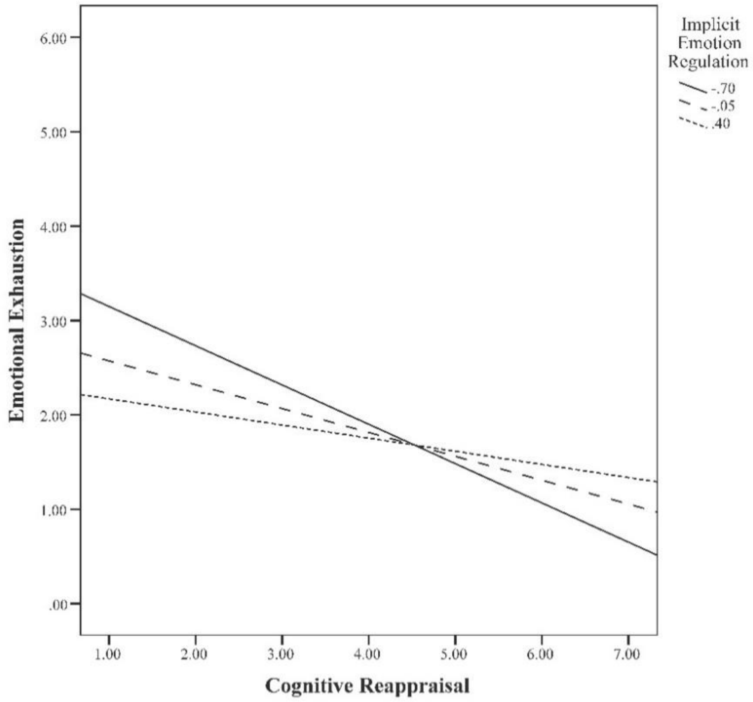


*Figure B1*. Visualization of the moderating effect of implicit attitudes towards emotion regulation on the relationship between cognitive reappraisal and emotional exhaustion (step 2).

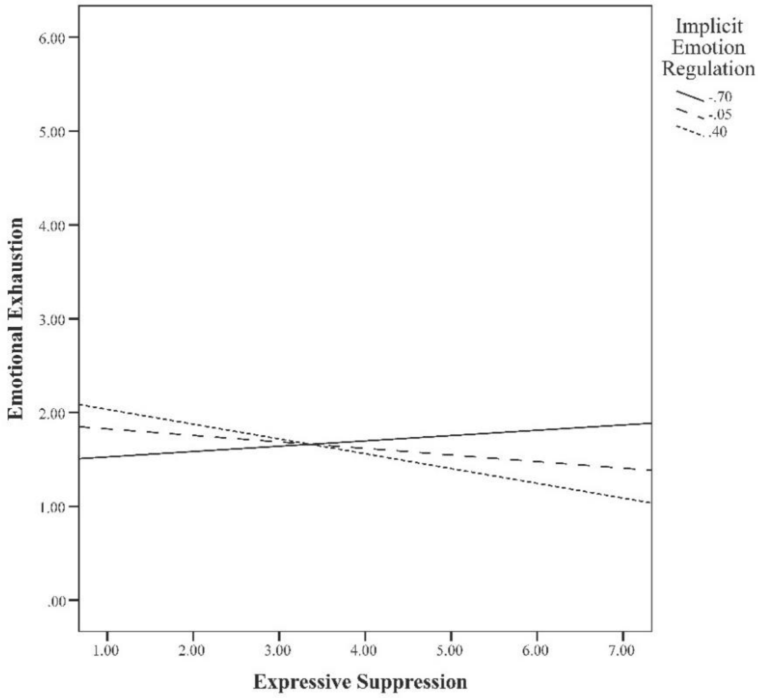


*Figure B2*. Visualization of the moderating effect of implicit attitudes towards emotion regulation on the relationship between expressive suppression and emotional exhaustion (step 2).
